# Two new species of the genus *Emertonia* Wilson, 1932 from Korean waters (Copepoda, Harpacticoida, Paramesochridae)

**DOI:** 10.3897/zookeys.718.19959

**Published:** 2017-12-04

**Authors:** Jinwook Back, Wonchoel Lee

**Affiliations:** 1 Department of Taxonomy and Systematics, National Marine Biodiversity Institute of Korea, Seocheon 33662, Korea; 2 Department of Life Science, College of Natural Sciences, Hanyang University, Seoul 04763, Korea

**Keywords:** Crustacea, Jeju Island, *Kliopsyllus*, Taxonomy, Yellow Sea

## Abstract

Two new species of the genus *Emertonia* were found from the west coast of Korea. The first new species, *E. koreana* **sp. n.**, is closely related to *E. acutifurcata*. However, the new species is clearly distinguished by the presence of two modified pinnate setae on the P5 baseoendopodal lobe. All body somites of the new species except for the last two urosomites have strongly developed hyaline frills forming quadrilateral lappets. The second new species, *E. simplex* **sp. n.**, superficially resembles *E. mielkei* in the structure of antennary exopod (with five setae), and the shape of P5. However, this new species differs from its congener mainly by having a caudal ramus 3.5 times as long as width, and P1 enp-2 with two claw-like setae. In addition, a key to the worldwide species of *Emertonia* is provided.

## Introduction

The family Paramesochridae consists of 13 genera and more than 150 species distributed worldwide. Within the family, the genus *Emertonia* Wilson, 1932 is seen to be the most species-rich genus. Despite the rich diversity, there are still many unidentified species to be regarded as new species within the genus (Plum and George 2009; Back and Lee 2014). According to Plum and George (2009), most of the species of Emertonia are discovered in the interstitial and coastal zone with an exception to four species found from the deep sea, *E. andeep* (Veit-Köhler, 2004), *E. diva* (Veit-Köhler, 2005), *E. minor* (Vasconcelos, Veit-Köhler, Drewes & Parreira dos Santos, 2009), and *E. schminkei* (Veit-Köhler & Drewes, 2009).

Kunz (1962) divided the family Paramesochridae into nine genera based on the segmentation, and setae formula of swimming legs. Although Kunz (1962) proposed the name *Kliopsyllus* with the generic diagnosis based on four species (*Leptopsyllus coelebs* Monard, 1928; *Paramesochra holsatica* Klie, 1929; *L. constrictus* Nicholls, 1935, and *P. major* Nicholls, 1939) and two sub-species (*P. holsatica varians* Kunz, 1951, and *P. constricata orotavae* Noodt, 1958), he failed to fix the type species for the genus. Huys (2009) claimed that *Emertonia* Wilson, 1932 is the replacement name for *Kliopsyllus*. To date, 48 species including six sub-species have been reported in *Emertonia*, and most species are found from sandy sediments.

In Korea, taxonomic studies on coastal benthic copepods are underway. Song et al. (2012) summarized the marine and brackish-water harpacticoids found in Korea. They reported a list including 88 marine and brackish-water harpacticoids belonging to 23 families (Song et al. 2012). Especially in the case of the family Paramesochridae, 11 species are found in the coastal sandy sediments (Back and Lee 2014; Back and Lee 2017). As a part of ongoing taxonomical study on the harpacticoid copepods, we aim to describe two new species of *Emertonia* sampled from sandy beaches in Korean Waters.

## Materials and methods

The sediment samples for *Emertonia koreana* sp. n. were collected from the Chulripo Beach in the west coast of the Korean peninsula. The sediment samples for *E. simplex* sp. n. were collected from a subtidal zone of Jeju Island. Samples were fixed with 5% buffered formalin and dissected specimens were mounted on several slides separately using lactophenol as mounting medium. Slides were sealed with transparent nail varnish. Observations of the specimens were carried out using an LEICA DM 6000 equipped with a drawing tube. Specimens were deposited in the Marine Biodiversity Institute of Korea (MABIK).

To prepare specimens for scanning electron microscope analysis (SU3500; Hitachi, in National Marine Biodiversity Institute of Korea), specimens were transferred to 100 % ethanol, dehydrated by t-BuOH freeze dryer (VFD-21S; Vacuum Device), mounted on stubs using double-sided tape, coated with gold-palladium, and then photographed.

The descriptive terminology was adopted from Huys et al. (1996). Abbreviations used in the text are:

**A1** antennule;

**A2** antenna;

**ae** aesthetasc;

**exp** exopod;

**enp** endopod;

**exp (enp)-1 (2, 3)** to denote the proximal (middle, distal) segment;

**P1–P6** first to sixth thoracopod;

**benp** baseoendopod.

## Systematics

### Order Harpacticoida Dana, 1846

#### Family Paramesochridae Lang, 1944

##### Genus *Emertonia* Wilson, 1932

###### Emertonia koreana sp. n.

Taxon classificationAnimaliaHarpacticoidaParamesochridae

http://zoobank.org/FE323D1F-32E2-412B-93DF-38AFF9B6A2BF

**Figure 1.**
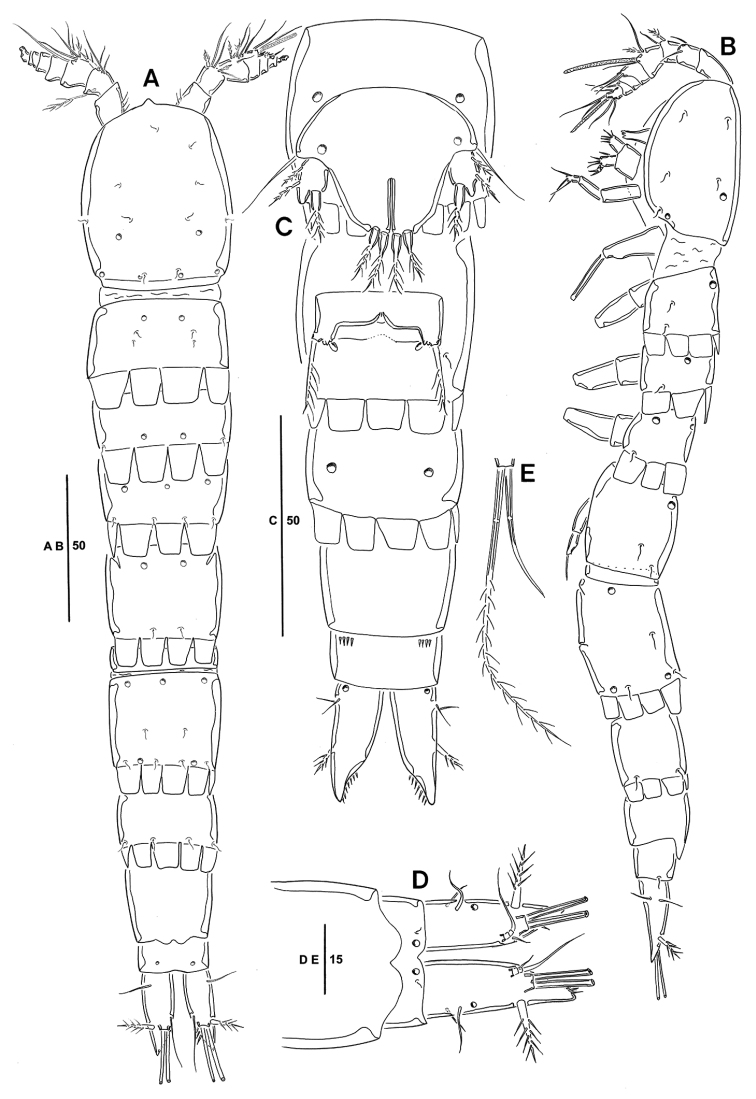
*Emertonia koreana* sp. n., holotype (♀). **A** habitus, dorsal **B** habitus lateral **C** urosome, ventral **D** caudal rami, dorsal **E** caudal seta IV and V. Scale bars are in µm.

**Figure 2.**
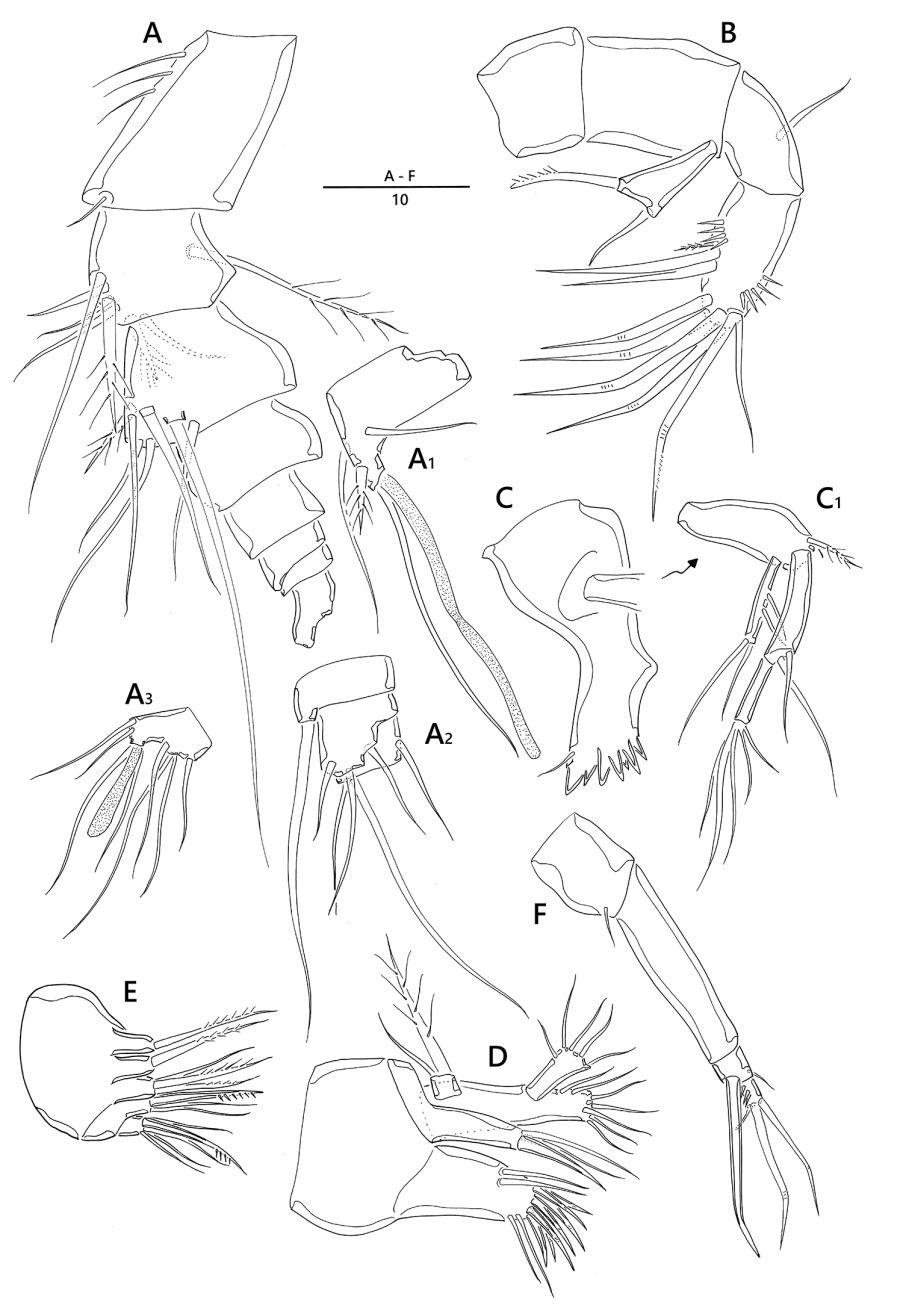
*Emertonia koreana* sp. n., holotype (♀). **A** antennule (**A_1_** fourth segment **A_2_** fifth, sixth, and seventh segments **A_3_** last segment) **B** antenna **C** mandible (**C_1_** plap) **D** maxillule **E** maxilla **F** maxilliped. Scale bar is in µm

**Figure 3.**
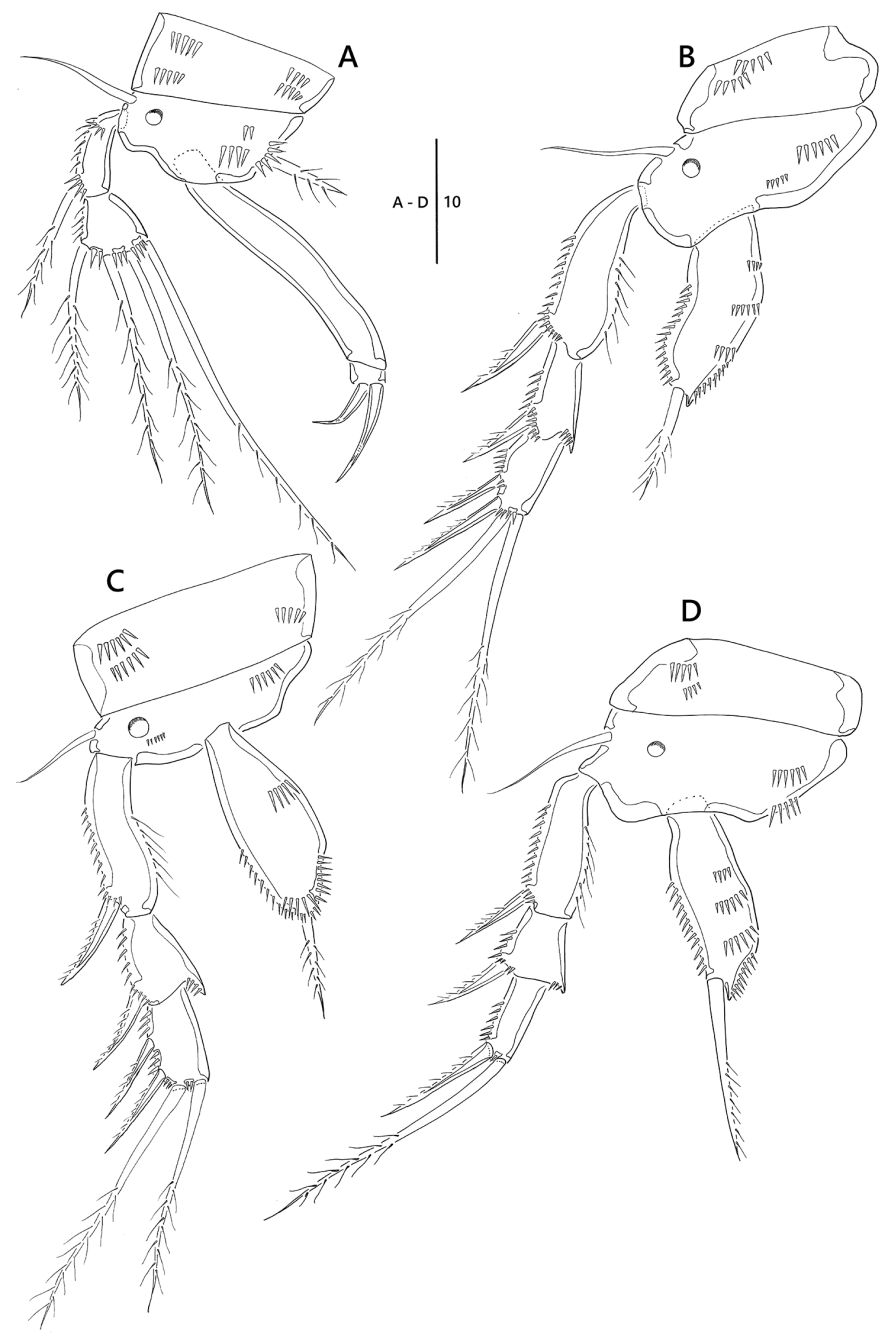
*Emertonia koreana* sp. n., holotype (♀). **A** P1 **B** P2 **C** P3 **D** P4. Scale bar is in µm.

**Figure 4.**
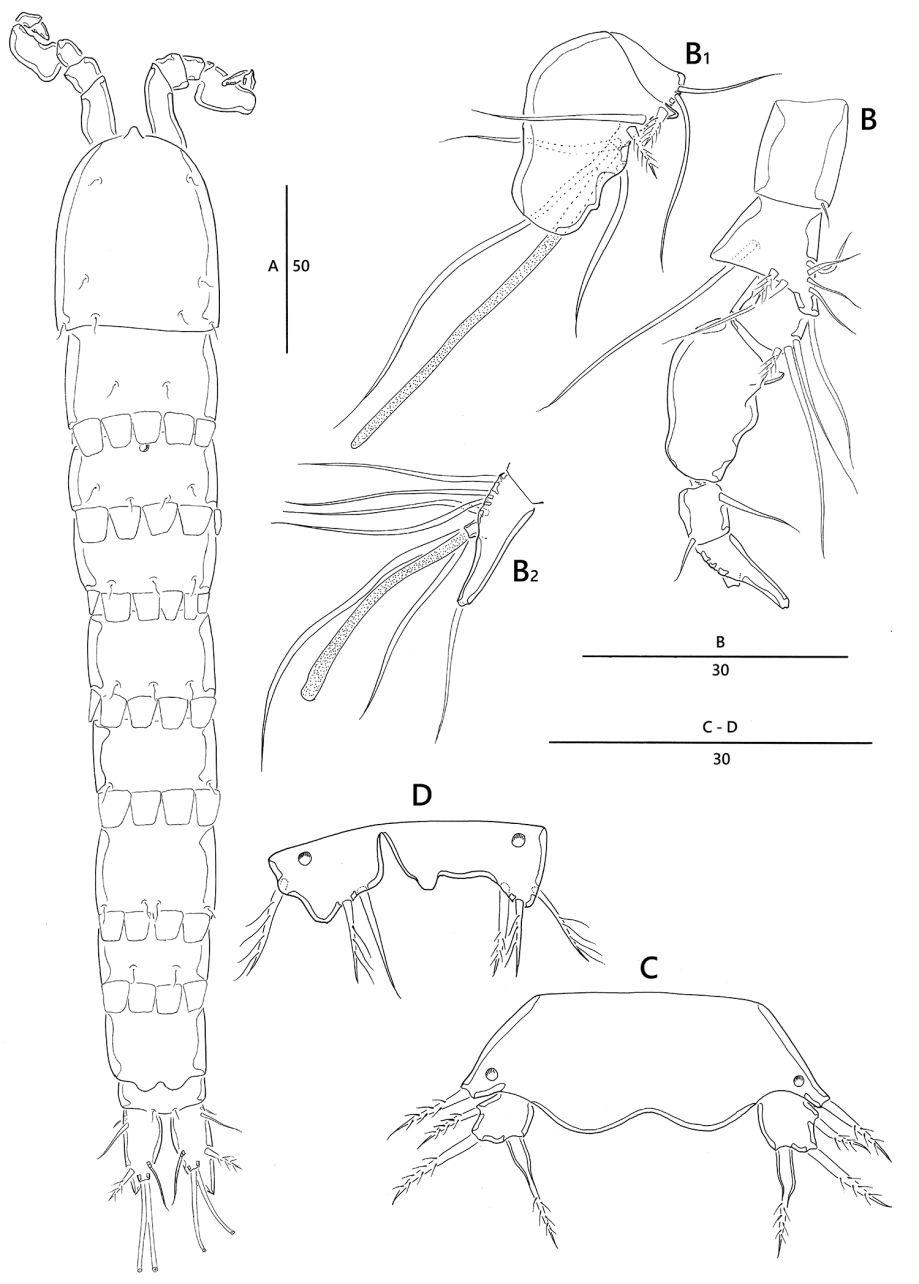
*Emertonia koreana* sp. n., (♂). **A** habitus, dorsal **B** antennule (**B_1_** fifth segment **B_2_** last segment) **C** P5 **D** P6. Scale bars are in µm.

**Figure 5.**
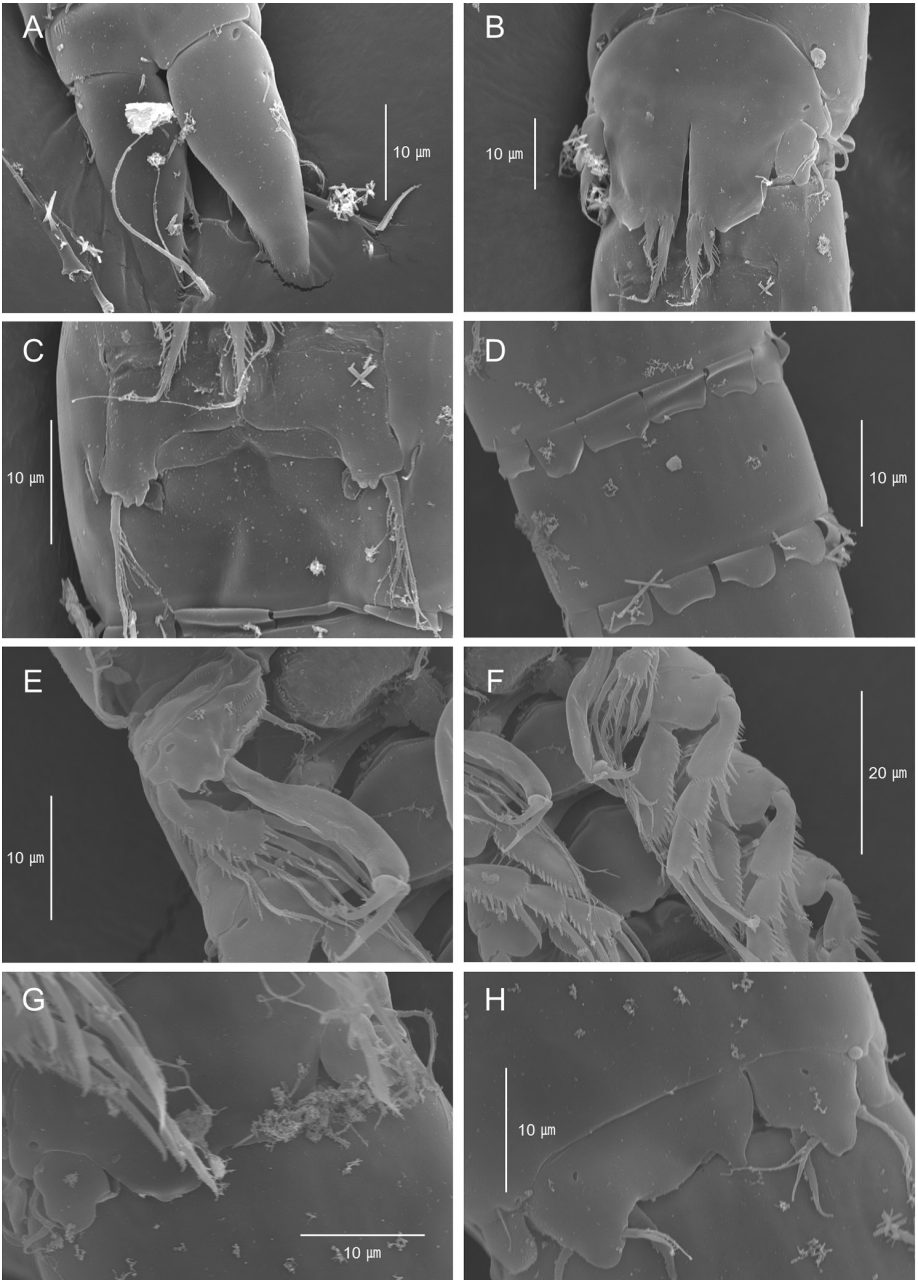
*Emertonia koreana* sp. n., SEM photographs. **A** caudal rami, ventral (♀) **B** P5 (♀) **C** P6 (♀) **D** sixth and seventh somites, ventral (♂) **E** P1 (♂) **F** P2 and P3 (♂) **G** P5 (♂) **H** P6 (♂).

Figs 1 , 2 , 3 , 4 , 5

####### Type locality.

The Chulripo Beach, intertidal zone in the west coast of Korea, Yellow Sea (36°48'11.46"N, 126°08'58"E) by sand rinsing collected by J. Back on 14 May 2010 (Back and Lee 2014, as *Emertonia* sp. 3)

####### Material examined.

Holotype 1♀ dissected on 4 slides (MABIK CR00241565). Paratypes: 1♂ on 3 slides (MABIK CR00241566), and 5 ♀♀ (MABIK CR00241570 – 00241574), 3 ♂♂ (MABIK CR00241567 – CR00241569) in 70 % ethanol. 1♀ and 1♂ dried, mounted on stub, and coated with gold-palladium for SEM.

####### Diagnosis.

Female P5 deeply divided into two parts in the center of both P5 baseoendopods. Two setae of baseoendopod swollen near the base. Innermost seta of P5 exopods somewhat swollen at base, similar to setae of baseoendopod. Urosomites armed with rectangular frills, except for the last two segments.

####### Description of female.

Body. Length 330 µm (n = 6, mean = 325 µm); largest width measured at cephalic shield; 55 µm; cylindrical, slightly depressed dorsoventrally; whole body very hyaline; sensilla and pores on dorsal surface as figured (Fig. 1A, B).

*Prosome* (Fig. 1A, B). Comprising cephalothorax, and three free pedigerous somites; cephalothorax bell-shaped, with sensilla and pores as figured; pleural areas weakly developed and posterolateral angles rounded; posterior margin smooth; somites bearing P2–P4 with strongly developed hyaline frills forming quadrilateral lappets (Fig. 1A–C).

*Urosome* (Fig. 1A–C) Genital somite and first abdominal somite completely fused forming genital double-somite; genital field located mid-ventrally at approximately half length of genital double-somite; copulatory pore presumably covered by P6; P6 (Figs 1C, 5C) represented by one plate with one uni-pinnate seta each side; penultimate somite with bilobed, smooth pseudoperculum; anal somite small, with two pores dorsally.

*Caudal ramus* (Figs 1D, E, 5A). Juxtaposed, approximately 2.8 times as long as greatest width, conical, distal margin acutely pointed; each ramus armed with seven setae; seta I small, bare, arising ventrally; seta II bare; setae III stout, ornamented with spinule-like elements; seta IV bare; seta V pinnate, longest; seta VI shortest, bare; setae IV–VII displaced onto dorsal surface of ramus; seta VII bi-articulate at base and arising from inner dorsal surface.

*Rostrum* (Fig. 1A). Triangular, ventrally directed, fused with cephalic shield, without sensilla.

*Antennule* (Fig. 2A). Eight-segmented; proximal segment longest and ornamented with a few long spinules along lateral margin; fourth segment (Fig. 2A_1_) forming sub-cylindrical process armed with one long slender seta fused basally to aesthetasc; sixth segment (Fig. 2A_2_) armed with one slender bare seta arising from ventral sub-cylindrical process; armature formula: 1 – [1 bare], 2 – [5 bare + 3 pinnate], 3 – [6 bare + 1 pinnate], 4 – [2 bare + 1 pinnate + (1 + ae)], 5 – [1 bare], 6 – [3 bare], 7 – [3 bare], 8 – [6 bare + acrothek]; apical acrothek (Fig. 2A_3_) consisting of short aesthetasc fused basally to two naked setae.

*Antenna* (Fig. 2B). Four-segmented, comprising coxa, basis, one-segmented exp, and two-segmented enp; coxa small and bare; basis without any surface ornamentation; exp unequal Y-shape with one bare and one uni-pinnate setae; enp-1 with one bare abexopodal seta; enp-2 armed with one pinnate spine, two spine-like setae laterally, four geniculate setae around distal margin, and one longest geniculate seta fused at base with one bare seta.

*Mandible* (Fig. 2C). Coxa with gnathobase bearing one bare seta at dorsal corner and seven teeth; palp (Fig. 2C_1_) biramous, comprising basis, one-segmented exp and two-segmented enp; basis widening distally, with one pinnate seta; exp with two lateral and two distal setae; enp-1 with two bare setae; enp-2 with five basally fused setae at apex.

*Maxillule* (Fig. 2D). Praecoxal arthrite well developed, with seven spines, two bare setae around distal margin, and two juxtaposed slender setae on anterior surface near outer margin; coxa fused with cylindrical endite, armed with two bare setae and one stout spine; basis fused with endite, armed with seven bare setae; exp one-segmented, small, with one bare and one pinnate setae; enp one-segmented, with five bare setae.

*Maxilla* (Fig. 2E). Syncoxa with three endites; proximal and second endites with one pinnate seta; third endite with one bare and two uni-pinnate setae; allobasis with one strong pinnate claw and two bare setae; enp one-segmented, with one stout spine and four bare setae.

*Maxilliped* (Fig. 2F) four-segmented, comprising syncoxa, basis and two-segmented enp; syncoxa with one bare seta distally; basis bare; enp-1 with one geniculate and one small setae; enp-2 small, with two geniculate and one bare setae around distal margin.

*P1* (Fig. 3A). Coxa ornamented with rows of spinules; basis with one pinnate inner seta and one bare outer seta, and ornamented with one pore near base of outer seta; enp 1.9 times as long as exp; exp two-segmented; exp-1 with one pinnate outer seta; exp-2 short, sub-quadrilateral, with three pinnate and one uni-pinnate setae; enp two-segmented; enp-1 long, bare; enp-2 small, with two short geniculate setae.

*P2, P3* (Fig. 3B, C). Coxa ornamented with rows of spinules; basis with one bare outer seta, one pore near base of exp, and rows of spinules as figured; exp three-segmented; exp-1 with one outer spine and ornamented with row of long spinules along inner margin; exp-2 with one outer spine, inner distal corner forming spinous projection; exp-3 with two outer spines and two pinnate setae; enp one-segmented, with one plumose apical seta.

*P4* (Fig. 3D). Coxa ornamented with two rows of spinules on anterior surface; basis with one bare outer seta and one pore; exp three-segmented; exp-1 and exp-2 with one outer spine; exp-3 with one outer spines and one pinnate apical seta; enp one-segmented with one apical seta.

**Armature formula as follows**:

**Table:** 

	**Exopod**	**Endopod**
P1	0.121	0.011
P2	0.0.112	010
P3	0.0.112	010
P4	0.0.011	010

*P5* (Figs 1C, 5B). Comprising medially fused benps and discrete exps; benp with one basal seta and ornamented with one pore; endopodal lobes elongated and separated by median cleft; each with two pinnate modified setae; exopod with two pinnate and one modified setae, and outer corner forming projection.

####### Description of male.

Body (Fig. 4A) length 320 µm (n = 4, mean = 315 µm); largest width measured at posterior margin of cephalic shield: 45 µm; general body shape and ornamentation as in female; except for last two urosomites, urosome somites present strongly developed hyaline frills from dorsal to venteral (Fig. 5D); additional sexual dimorphism in A1, P5, and P6.

*Antennule* (Fig. 4B). Seven-segmented, short, robust, subchirocer; fifth-segment (Fig. 4B_1_) swollen, largest, forming sub-cylindrical process with one long slender seta fused basally to aesthetasc. Armature formula: 1 – [1 bare], 2 – [7 bare + 1 pinnate], 3 – [2 bare + 1 pinnate], 4 – [2 bare], 5 – [3 bare + 2 pinnate + (1 + ae)], 6 – [2 bare], 7 – [5 bare + acrothek], acrothek (Fig. 4B_2_) consisting of aesthetasc and two bare setae.

*legs* P1–P4 shape and setae formulae as in female (Fig. 5E, F)

*P5* (Figs 4C, 5G). Comprising medially fused benp and discrete exp; benp with one basal seta; endopodal lobes weakly developed, without any element; exopod small, with two pinnate outer and one modified inner setae.

*P6* (Figs 4D, 5H). Asymmetrical; each P6 with one outer and two inner setae, ornamented with one pore.

####### Etymology.

The species name refers the type locality of new species, Republic of Korea.

####### Remarks.

The new species *Emertonia koreana* sp. n. is closely related with *E. acutifurcata* (Mielke, 1985). They share similar shape of caudal ramus. *E. koreana* sp. n. and *E. acutifurcata* only have sub-triangular caudal ramus. Within the genus *Paramesochra*, similar morphology of caudal ramus is observed in *P. acutata acutata* Klie, 1935, *P. acutata hawaiiensis* Kunz, 1981, and *P. taeana* Back & Lee, 2010. They also have same setal formula of P1–P5. *E. koreana* sp. n. can be easily distinguished from those species based on the following unique characteristics: 1) female P5 is deeply divided into two parts in the center of both P5 baseoendopods. 2) Two setae at the end of baseoendopod are swollen near the base. In addition, the base of the innermost seta of P5 exopods is swollen, similar to setae of baseoendopod. 3) There are rectangular frills, except for the last two segments of urosomite. This structure is similar to that of *P. taeana*, but has not been reported in the genus *Emertonia* yet.

###### Emertonia simplex sp. n.

Taxon classificationAnimaliaHarpacticoidaParamesochridae

http://zoobank.org/2E7AB618-93AA-4942-AC8C-B3DB5183CFF9

**Figure 6.**
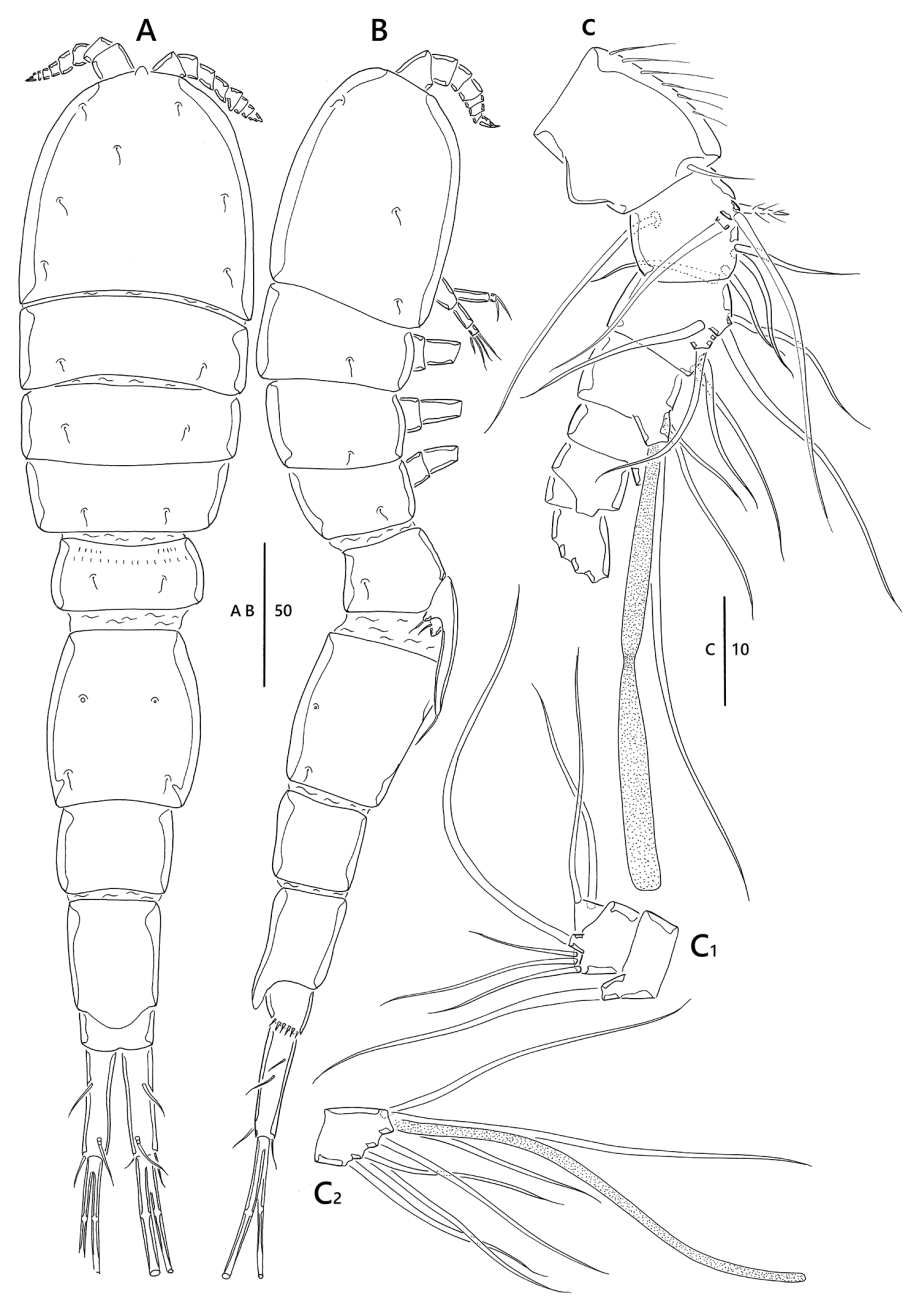
*Emertonia simplex* sp. n., (♀) **A** habitus, dorsal **B** habitus, lateral **C** antennule (**C_1_** fifth, sixth, and seventh segments **C_2_** last segment). Scale bars are in µm.

**Figure 7.**
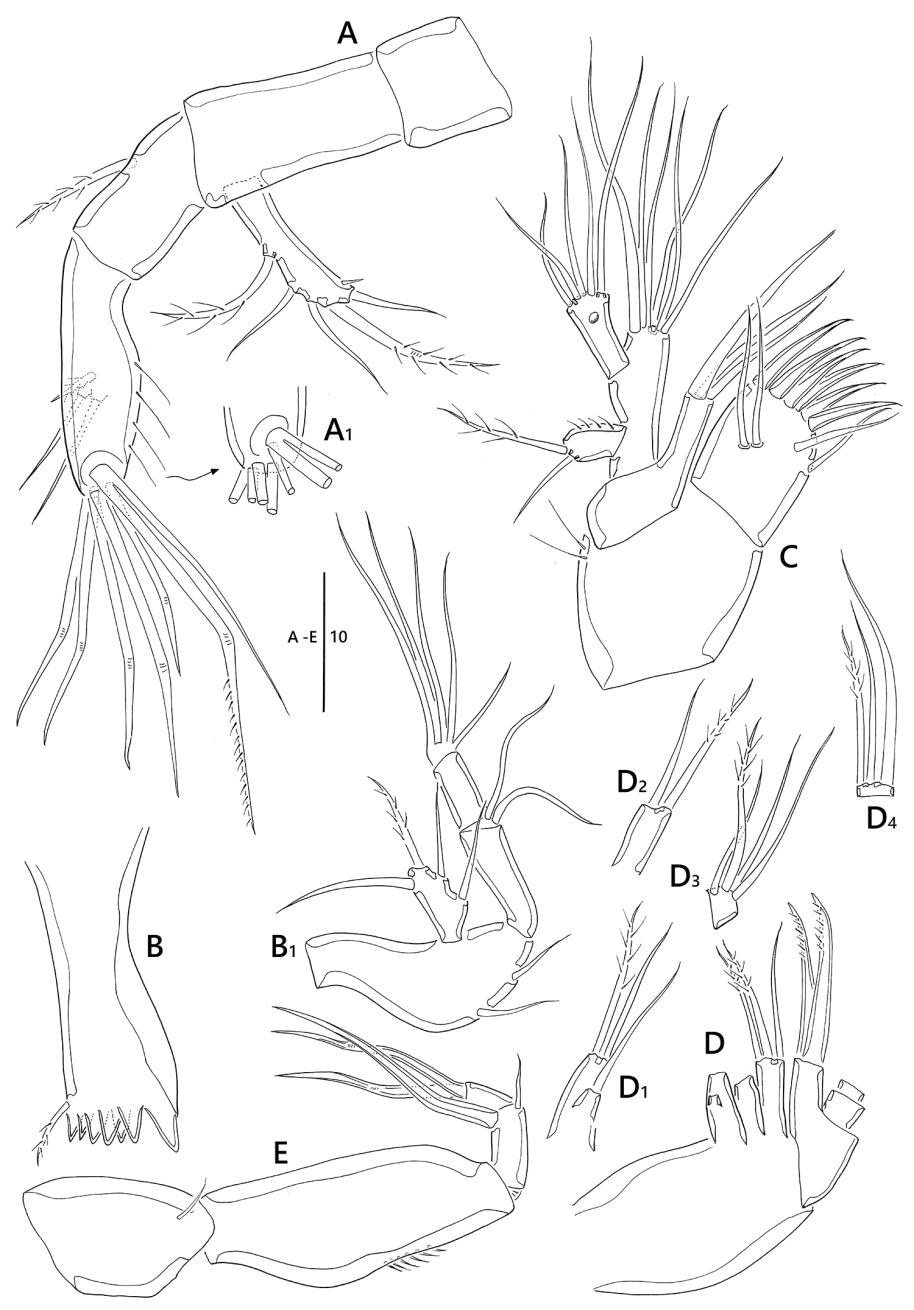
*Emertonia simplex* sp. n., (♀). **A** antenna (**A_1_** end of second segment in antenna endopod) **B** mandible (**B_1_** palp) **C** maxillule **D** maxilla (**D_1_** first endite **D_2_** second endite **D_3_** first segment of endopod **D_4_** second segment of endopod) **E** maxilliped. Scale bar is in µm.

**Figure 8.**
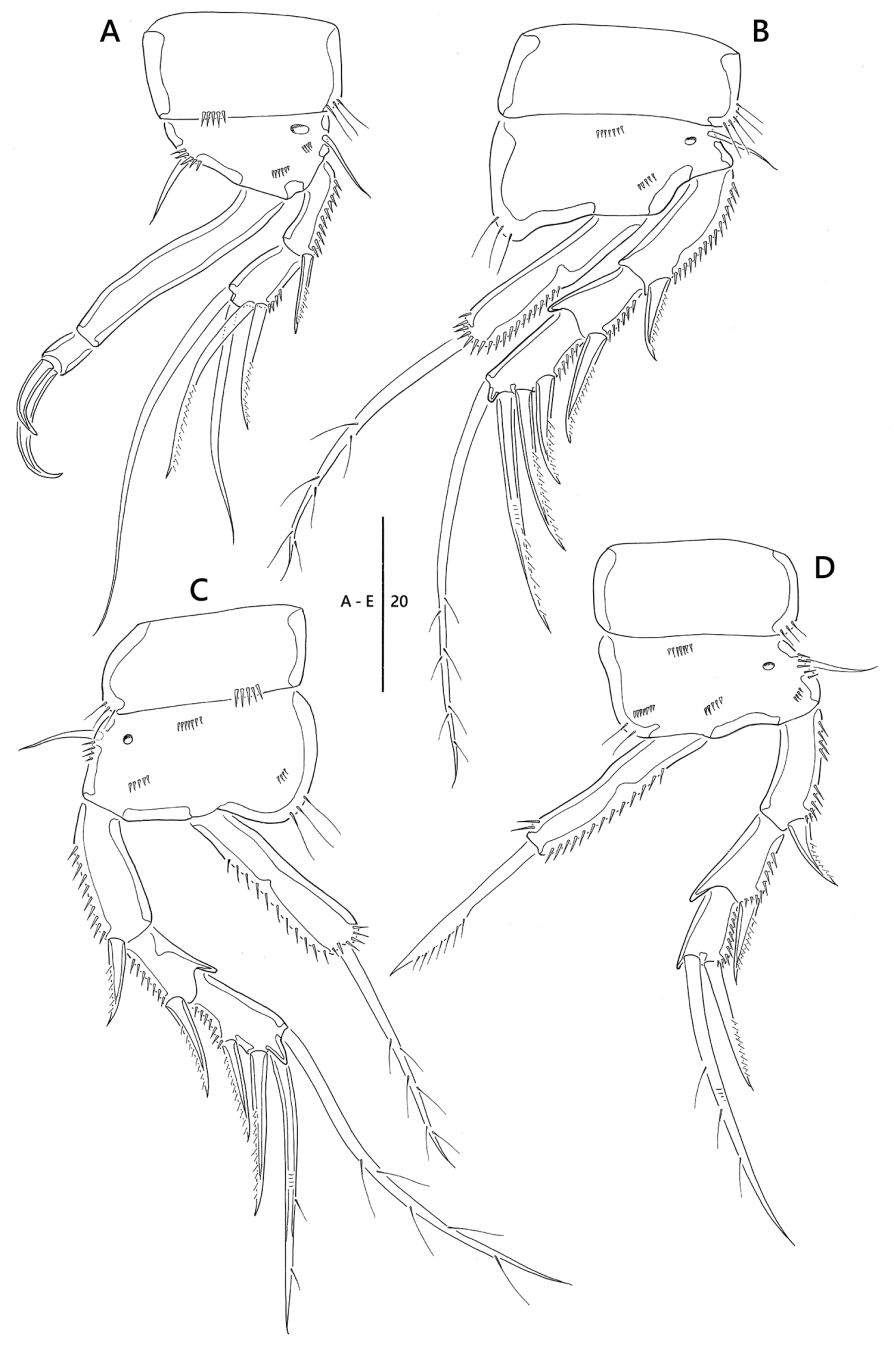
*Emertonia simplex* sp. n., (♀). **A** P1 **B** P2 **C** P3 **D** P4. Scale bar is in µm.

**Figure 9.**
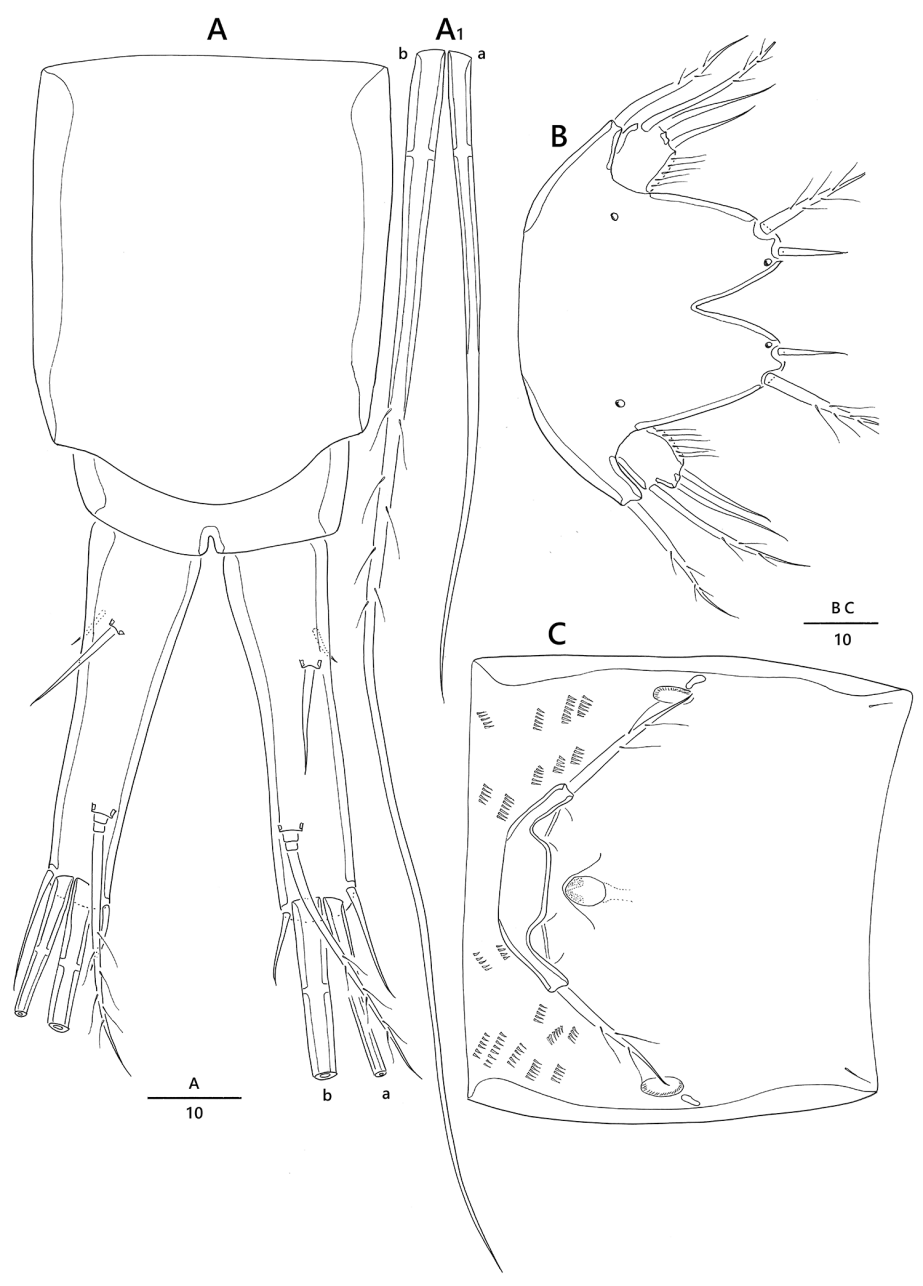
*Emertonia simplex* sp. n., (♀). **A** last two segments of urosomite and caudal rami **B** P5 **C** P6 and genital field. Scale bars are in µm.

**Figure 10.**
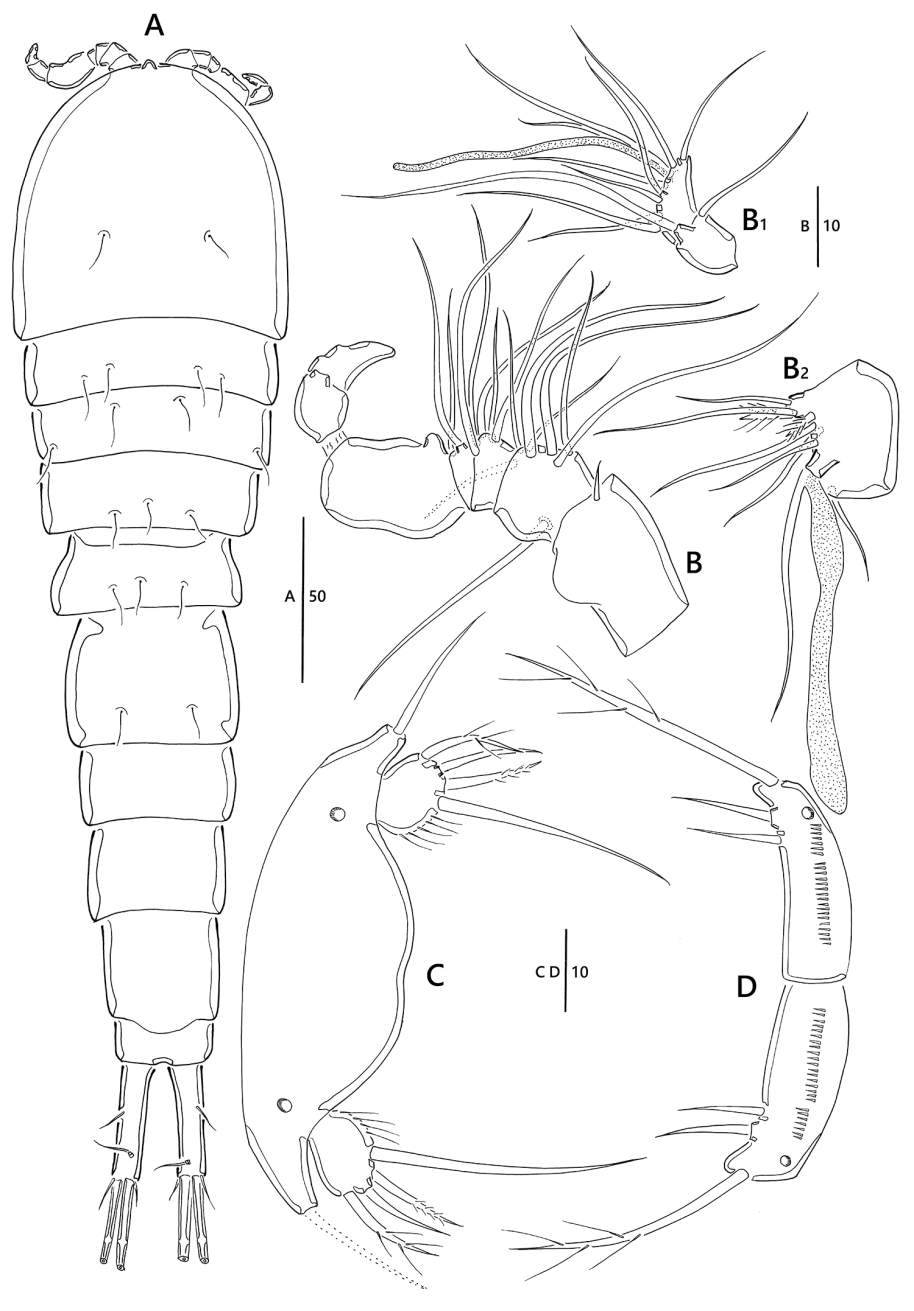
*Emertonia simplex* sp. n., (♂). **A** habitus, dorsal **B** antennule (**B_1_** last two segments **B_2_** fifth segment) **C** P5 **D** P6. Scale bars are in µm.

**Figure 11.**
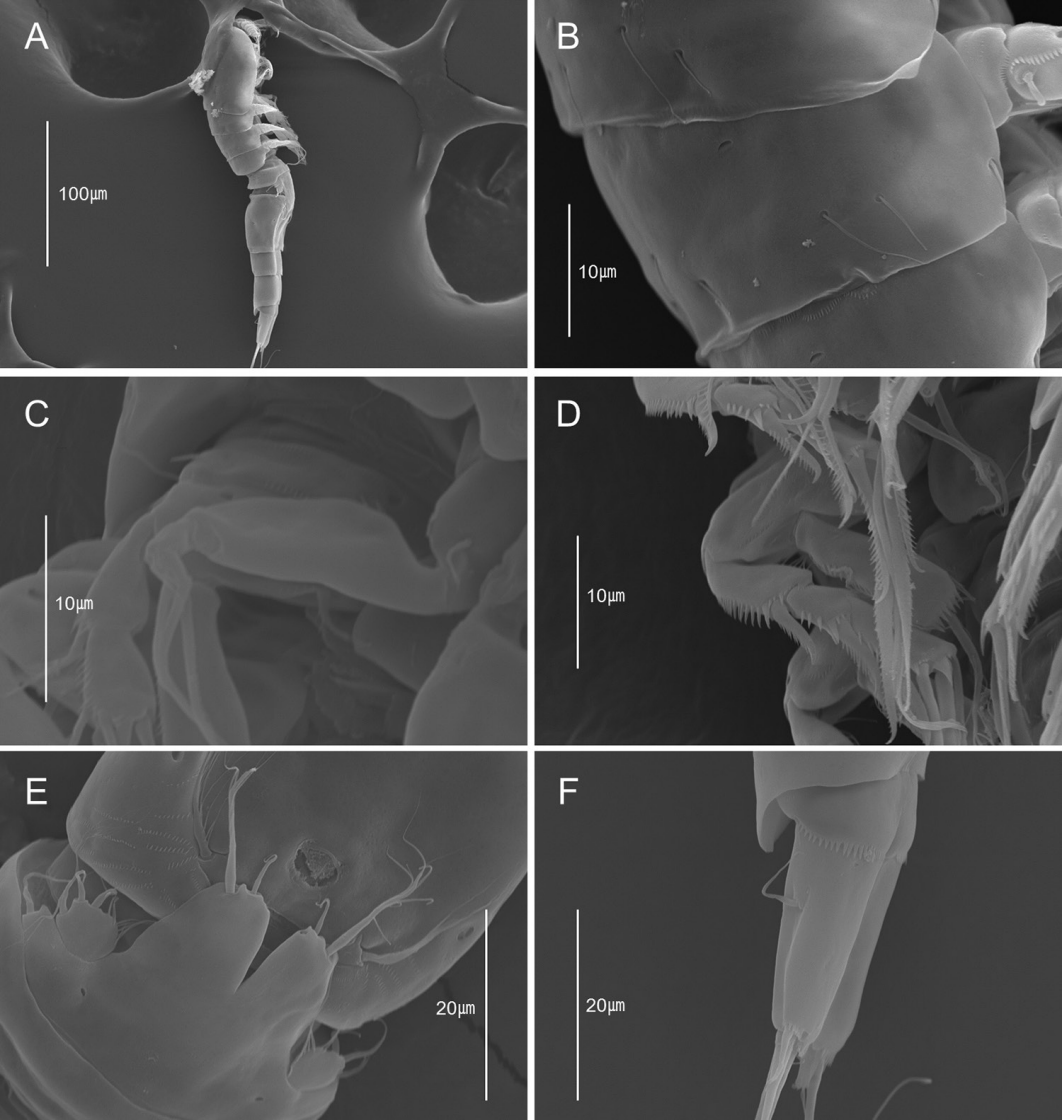
*Emertonia simplex* sp. n. (♀), SEM photographs. **A** habitus, lateral **B** first and second segment, lateral **C** P1 **D** P3 **C** P3 **E** P5 **F** caudal rami, lateral.

Figs 6 , 7 , 8 , 9 , 10 , 11

####### Type locality.

**A** subtidal zone near the Seogwipo Port in Jeju Island, Korea (33°13'33"N, 126°34'39"E), and sampled by using a grab (surface area: 0.1 m^2^) on a fishing boat (Back and Lee 2014, as *Emertonia* sp. 2), depth 15–20 m, sand.

####### Material examined.

Holotype 1♀ dissected on 7 slides (MABIK CR00241575), and paratypes: 1♂ on 5 slides (MABIK CR00241576). Additional paratypes represented by 3 ♀♀ (MABIK CR00241577 ~ CR00241579) and 2 ♂♂ (MABIK CR00241580, CR00241581) in 70 % ethanol. 2♀♀ dried, mounted on stub, and coated with gold-palladium for SEM. All samples were collected from the type locality by J. Back on 4 June 2010.

####### Diagnosis.

*Emertonia simplex* sp. n. with four setae at P5 exopod in male, and one short Inner seta at P5 baseoendopod in female. Caudal rami rectangular, approximately 3.8 times as long as its width. Body armed with long dorsal sensilla.

####### Description of female.

Body cylindrical, slightly depressed dorsoventrally (Figs 6A–B, 11A), with long sensilla (Fig. 11B); total body length, 390 µm (n = 7, mean = 376 µm); largest width (85 µm) measured at posterior margin of cephalic shield; body somites without hyaline frills forming quadrilateral lappets.

*Prosome* (Fig. 6A, B). Comprising cephalothorax, and three free pedigerous somites; Cephalothorax bell-shaped, with several sensilla; pleural areas weakly developed, posterolateral angles rounded.

*Urosomites* (Fig. 6A, B). Gradually tapering posteriorly; genital somite and third urosomite completely fused forming genital double-somite (Figs 9C, 11E); genital field located in proximal half of genital double-somite, with copulatory pore positioned medially, and two pores; P6 (Figs 9C, 11E) represented by narrow transverse plate, each side armed with one pinnate seta; anal somite (Fig. 9A) without anal operculum, but with rounded pseudoperculum arising from penultimate somite.

*Caudal rami* (Figs 9A, 11F). Rectangular, approximately 3.2 times as long as wide; with seven setae; setae III–VI located around distal margin of ramus; seta I small, bare, arising laterally; seta II bare; seta III cylindrical, bare; seta IV well developed, bare, seta V longest, pinnate in middle; seta VI bare; dorsal seta VII bi-articulate at base, bipinnate in middle.

*Rostrum* (Fig. 6A) small, with rounded tip, fused with cephalothorax; without sensilla.

*Antennule* (Fig. 6C) slender, eight-segmented; proximal segment with row of long spinules along anterior margin and blunt process on lateral margin; fourth segment with sub-cylindrical process bearing one bare seta fused basally to aesthetasc; fifth segment with sub-cylindrical process with one bare seta (Fig. 6C_1_); armature formula: 1 – [1], 2 – [7 bare + 1 spinulose], 3 – [6 bare], 4 – [2 bare + (1 + ae)], 5 – [1 bare], 6 – [2 bare], 7 – [4 bare], 8 – [5 bare + (2 + ae)]; apical acrothek consisting of one apical aesthetasc and two basally fused bare setae (Fig. 6C_2_).

*Antenna* (Fig. 7A). Coxa and basis without surface ornamentation; exp one-segmented, with two pinnate and three bare setae, and one spinule near outer distal corner; enp two-segmented; enp-1 with one pinnate seta, without surface ornamentation; lateral armature of enp-2 consisting of two bare setae, one pinnate seta, and long spinules along outer margin; distal armature of enp-2 (Fig. 7A_1_) consisting of six geniculate and one bare setae (one long bare seta fused at base to largest geniculate seta).

*Mandible* (Fig. 7B). Coxa well developed; gnathobase with seven blunt teeth and one small pinnate seta at dorsal corner; palp biramous (Fig. 7B_1_), basis elongate, with two bare setae; exp one-segmented, with one pinnate and three bare setae; enp two-segmented, enp-1 1.7 times as long as enp-2, enp-1 with two bare setae; enp-2 with five setae fused at base.

*Maxillule* (Fig. 7C). Praecoxa subquadrate, with two long spinules; arthrite well developed, with six strong spines and two bare lateral setae, and two juxtaposed setae on surface; coxa with fused endite and three bare setae; basis fused with endites, with six setae; exp one-segmented, with one pinnate and one bare setae, and ornamented with row of spinules along inner margin; enp one-segmented, longer than exopod, with five bare setae around distal margin and one pore sub-distally.

*Maxilla* (Fig. 7D). Syncoxa armed with three endites; first endite (Fig. 7D_1_) bilobed, with one pinnate and two bare setae; second endite (Fig. 7D_2_) with one pinnate seta and one bare setae; distal endite with two pinnate and one bare setae; allobasis with two uni-pinnate stout setae on distal margin; enp two-segmented; enp-1 rectangular, with one pinnate seta near base, two bare and one pinnate setae; enp-2 with one pinnate and two bare setae along distal margin.

*Maxilliped* (Figs 7E, 11C) four-segmented; syncoxa with one bare seta; basis and ornamented with row of spinules along outer margin; enp two-segmented; enp-1 with one small bare seta laterally and one stout claw on distal margin; enp-2 with two geniculate setae.

*P1* (Fig. 8A). Coxa and basis with spinules as figured; the latter with one bare outer and one bare inner setae; exp two-segmented; exp-1 longer than exp-2, the former with row of spinules along outer margin and one uni-pinnate outer spine; exp-2 with two uni-pinnate and two bare setae; enp approximately 1.8 times as long as exp; enp-1 elongate, bare, approximately five times as long as enp-2; enp-2 small, slightly longer than wide, with two claw-like setae.

*P2, P3* (Figs 8B, C, 11D). Coxa with row of spinules on outer distal corner; basis with one bare outer seta, one pore near base of outer seta; exp three-segmented; exp-1 and exp-2 with one uni-pinnate spine; exp-3 with two uni-pinnate outer spines, one stout apical seta, and one pinnate seta; enp one-segmented with one pinnate apical seta.

*P4* (Fig. 8D). Coxa ornamented with one row of spinules on outer distal corner; basis with one bare outer seta, one pore near base of outer seta; exp three-segmented; exp-1 and exp-2 with one uni-pinnate outer spine; exp-3 with one uni-pinnate outer spine and one apical seta; enp one-segmented, with one modified seta.

**Armature formula as follows**:

**Table:** 

	Exopod	Endopod
P1	0.121	0.011
P2	0.0.112	010
P3	0.0.112	010
P4	0.0.011	010

*P5* (Figs 9B, 11E) with medially fused benps and discrete exps; benp with one pinnate outer basal seta; endopodal lobes well-developed, rounded, median cleft reaching at distal margin of exp, with one shorter inner and one longer outer setae; exp well developed, with one pinnate outer seta and two bare inner setae, and ornamented with a row of long spinules along inner margin.

####### Description of male.

Smaller than female, body length 345 µm (n = 3, mean = 344 µm) (Fig. 10A); largest width (80 µm) measured at posterior margin of cephalic shield; general body shape and ornamentation as in female except for separation of genital somite; additional sexual dimorphism in antennule, A1, P5, and P6.

*Antennule* (Fig. 10B). Seven-segmented, subchirocer; fifth segment (Fig. 10B_2_) swollen, largest; aesthetascs on fifth and seventh segments (Fig. 10B_1_); armature formula: 1 – [1 bare], 2 – [8 bare], 3 [5 –bare], 4 – [2 bare], 5 – [6 bare + 2 spinulose + (1 +ae)], 6 – [2 bare], 7 – [7 bare + (2 + ae)]; apical acrothek consisting of apical aesthetasc and two basally fused bare setae.

*P5* (Fig. 10C). Benp confluent, forming large transverse plate, with one bare outer basal seta and one pore on either side; exp ovate bearing setules on inner margin, with two pinnate and two bare setae, innermost longest.

*P6* (Fig. 10D). Clearly distinct medially, each P6 with one outer pinnate and two bare inner setae.

####### Etymology.

The species name refers to the simple somites without hyaline frills forming quadrilateral lappets.

####### Remarks.

The second new species, *Emertonia simplex* sp. n., shares the general characteristics of other species like *E. holsatica holsatica* (Klie, 1929) and *E. major* (Nicholls, 1939), including segmentation and setal formula of swimming legs, rectangular caudal rami, and a well-developed P5 baseoendopod and separated exopod. However, *E. simplex* sp. n. has a combination of all the following characteristics: 1) exopod of antenna has five setae. This characteristic is found in eleven species of *Emertonia*, for instance *E. regulexstans* (Mielke, 1984b), and *E. diva* (Veit-Köhler, 2005), and 2) Two claw-like setae are present on the second segment of P1, which can be found in *E. brevicaudata* (Kornev and Chertoprud, 2008), *E. californica* (Kunz, 1981), *E. insularis* (Kunz, 1981), *E. holsatica* s. str., *E. longifurcata* (Scheibel, 1975), and *E. unguiseta* (Mielke, 1984). Of these, *E. unguiseta* is the species closest to *E. simplex* sp. n. since they share all of the characteristics mentioned above. However, differences between *E. simplex* sp. n. and *E. unguiseta* are as follows: 1) *E. simplex* sp. n. has four setae at P5 exopod in male, whereas *E. unguiseta* bears only three setae, 2) the new species has a shorter inner seta at P5 baseoendopod in female, whereas *E. unguiseta* bears two subequal setae, 3) the length of caudal rami is approximately 3.8 times as long as its width, whereas that of *E. unguiseta* is 3.2 times of the width. In addition, the new species has long dorsal sensilla. The major morphological characteristics of the genus *Emertonia*, including the two new species were summarized in Table 1.

**Table 1.** Species list and morphological comparison of the species in genus *Emertonia* Wilson, 1932 based on female.

Species	A1	A2	P1			P2		P3			P4				P5		Caudal rami
	number of seg.	exp setae	exp-1	exp-2	enp-2	exp-2	exp-3	exp-2	exp-3	enp-1	exp-2	exp-3	enp-1	enp-2	exp	benp	shape
															setae	setae	width:lenth
*gracilis*-group																	
*E. gracilis* Wilson, 1932	8	3	023	·	011	0	011	0	011	010	0	011	010		3	2	square
																	1:2
*E. pseudogracilis* Krishnaswamy, 1957	7	2	122	·	011	0	012	0	012	010	0	011	010		3	1	square
																	1:2.5
*laurentica-group*																	
*E. laurentica* (Nicholls, 1939)	7	?	0	121	011	221	·	221	·	010	112	·	010		3	2	square
																	1:2
*andeep-group*																	
*E. andeep* (Veit-Köhler, 2004)	8	5	0	121	011	0	112	0	112	110	0	011	1	011	3	2	square
																	1:4
*E. minor* (Vasconcelos, Veit-Köhler, Drewes & Parreira dos Santos, 2009)	7	4	0	121	011	0	112	0	112	010	0	011	0	010	?	?	square
																	1:5
*coelebs*-group																	
*E. coelebs* (Monard, 1935)	7	4	0	121	011	0	112	0	112	010	1	011	110	·	3	?^1^	square
																	1:5
*E. psammophila* (Noodt, 1964)	8	3	0	121	011	0	112	0	112	010	1	011	010	·	3	2	square
																	1:2.5
*E. furcavaricata* Kunz, 1974	8	?	0	121	011	1	112	1	112	010	1	011	010	·	3	2	square
																	1:2.5
*E. atlantica* (Kunz, 1983)	7	3	0	121	011	0	112	0	112	010	1	011	110	·	3	1	square
																	1:3
*holsatica*-group																	
*E. holsatica holsatica* (Klie, 1929)	7	4(5)^2^	0	121	011	0	112	0	112	010	0	011	010	·	3	2	square
																	1:2
*E. holsatica varians* (Kunz, 1951)	7	5	0	121	011	0	112	0	112	010	0	011	110	·	3	2	square
																	1:3.5
*E. holsatica longicaudata* (Galhano, 1970)	7	2(3)^3^	0	121	011	0	112	0	112	010	0	011	010	·	3	2	square
																	1:3.5
*E. constricta constricta* (Nicholls, 1935)	7	2(1)^4^	0	121	011	0	012	0	012	010	0	012 (011)^4^	010	·	3	?	square
																	1:3
*E. constricta orotavae* (Noodt, 1958)	7	2(3)^5^	0	121	011	0	012	0	012	010	0	011	010	·	3	2	square
																	1:2
*E. constricta pacifica* (Mielke, 1984a)	8	4	0	121	011	0	012	0	012	010	0	011	010	·	3	2	square
																	1:3
*E. constricta egyptica* (Mittwally & Montagna, 2001)	8	4	0	121	011	0	012	0	012	010	0	011	010	·	3	2	square
																	1:4
*E. major* (Nicholls, 1939)	9	3	0	121	011	0	112	0	012	010	0	011	010	·	3	2	square
																	1:3
*E. pygmaea* (Nicholls, 1939)	7	3	0	121	011	0	112	0	112	010	0	011	010	·	5	2	square
																	1:2
*E. longisetosa* (Krishnaswamy, 1951)	7	2	0	121	011	0	112	0	112	010	0	011	010	·	3	2	square
																	1:3
*E. arenicola* (Krishnaswamy, 1957)	7	2	0	121	011	0	112	0	112	010	0	011	010	·	3	2	square
																	1:3
*E. capensis* Krishnaswamy, 1957	7	1	0	121	011	0	012	0	012	010	0	011	010	·	3	0	square
																	1:2
*E. minuta* Krishnaswamy, 1957	7	3	0	122	011	0	012	0	012	010	0	011	010	·	3	0	square
																	1:2
*E. enalia* (Krishnaswamy, 1957)	8	1	0	022	011	0	112	0	112	010	0	011	010	·	3	2	square
																	1:4(?)^6^
*E. wilsoni* (Krishnaswamy, 1957)	7	3	0	121	011	0	112	0	112	010	0	011	010	·	3	2	square
																	1:3
*E. pontica* (Serban, 1959)	9	3	?	?	?	?	?	?	?	?	?	?	?	·	3	2	square
																	1:3.5
*E. perharidiensis* (Wells, 1963)	7	4	0	121	011	0	112	0	012	010	0	011	110	·	3	2	square
																	1:5
*E. psammobionta* (Noodt, 1964)	7	3	0	121	011	0	112	0	112	010	0	011	010	·	3(2)	?	square
																	1:2
*E. idiotes* (Wells, 1967)	6	3	0	121	011	0	112	0	112	010	0	111	010	·	3	2	square
																	1:2.5
*E. paraholsatica* (Mielke, 1975)	7	4	0	121	011	0	112	0	112	010	0	011	010	·	3	2	square
																	1:3
*E. longifurcata* (Scheibel, 1975)	7	4	0	121	011	0	112	0	112	010	0	011	010	·	3	2	square
																	1:2.5
*E. spiniger spiniger* (Wells, Kunz & Rao, 1975)	7	5	0	022	020	0	112	0	112	010	0	011	110	·	3	2	square
																	1:6.5
*E. spiniger ornata* (Kunz, 1981)	7	5	0	022	020	0	112	0	112	010	0	011	110	·	3	2	square
																	1:9
*E. masryi* (Bodin, 1979)^7^	8	2	0	121	011	0	012	0	012	010	0	011	010	·	3	3	square
																	1:2
*E. californica* (Kunz, 1981)	7	3	0	121	011	0	112	0	112	010	0	020	010	·	3	2	square
																	1:3
*E. debilis* (Kunz, 1981)	8	3	0	121	011	0	112	0	112	010	0	011	010	·	3	2	square
																	1:2
*E. insularis* (Kunz, 1981)	8	4	0	121	011	0	112	0	112	010	0	011	010	·	3	2	square
																	1:3
*E. miguelensis* (Kunz,1983)	?	3	0	121	011	0	112	0	112	010	0	011	010	·	?	?	square
																	1:3.5
*E. panamensis* (Mielke, 1984a)	8	2	0	121	011	0	112	0	112	010	0	111	010	·	3	1	square
																	1:2
*E. regulexstans* (Mielke, 1984b)	8	5	0	121	011	0	112	0	112	010	0	011	010	·	3	2	square
																	1:2.3
*E. similis* (Mielke, 1984b)	8	5	0	121	011	0	112	0	112	010	0	011	010	·	3	2	square
																	1:3
*E. unguiseta* (Mielke, 1984b)	8	5	0	121	011	0	112	0	112	010	0	011	010	·	3	2	square
																	1:3.2
*E. acutifurcata* (Mielke, 1985)	?	4	0	121	011	0	112	0	112	010	0	011	010	·	3	2	subtriangular
																	1:4
*E. chilensis* (Mielke, 1985)	8	4	0	121	011	0	112	0	112	010	0	011	010	·	3	2	square
																	1:2
*E. diva* (Veit-Köhler, 2005)	8	5	0	121	011	0	112	0	112	010	0	011	011	·	3	2	square
																	1:5.5
*E. brevicaudata* (Kornev & Chertoprud, 2008)	7	4	0	121	011	0	112	0	112	010	0	011	010	·	3	2	square
																	1:1.6
*E. schminkei* (Veit-Köhler & Drewes 2009).	8	5	0	121	011	0	112	0	112	010	0	011	010	·	3	2	square
																	1:9
*E. clausi* Pointner & Veit-Köhler, 2013	7	5(6)^8^	0	121	010	0	112	0	112	010	0	011	010	·	3	2	square
																	1:5.5
*E. ingridae* Pointner & Veit-Köhler, 2013	8	5	0	121	010	0	112	0	112	010	0	011	010	·	3	2	square
																	1:4.5
*E. koreana* sp. n. (This study)	8	2	0	121	011	0	112	0	112	010	0	011	010	·	3	2	subtriangular
																	1:3
*E. simplex* sp. n. (This study)	8	5	0	121	011	0	112	0	112	010	0	011	010	·	3	2	square
																	1:3.8

^1^ Chappuis (1954) described P5 benp with two setae, however we doubt he described a different species instead of *E. constricta constricta*. In contrast to description of Nicholls (1935) and Marinov (1971), Chappuis (1954) described that the innermost seta of P5 exp is the longest among three setae.

^2^ Mielke(1975) illustrated A2 exp with five setae.

^3^ Galhano (1970) described exp of A2 with two setae in the manuscript but with three in the figure.

^4^ Marinov (1971) illustrated A2 exp with one seta and P4 exp-3 with two elements.

^5^Though Masry (1970) supported the redescription of *E. constricta orotavae*, Masry’s material had these differences as follow: A2 exopod with three setae (Noodt’s species has two setae), baseoendopodal lobe with two bare equal length setae (Noodt’s species has one longer inner and one shorter inner pinnate setae, caudal seta III ornamented with strong spinules (Noodt’s species has a pinnate seta), and the innermost seta of P1 enp-2 bare (Noodt’s species has a uni-pinnate seta).

^6^ Krishnaswamy (1957) illustrated the habitus with very small caudal ramus. It is very difficult to calculate the ratio of width:length.

^7^The characters of *E. masryi* (Bodin, 1979) were based on *Kliopsyllus minutus* in Masry (1970).

^8^Pointner et al. (2013) described exp of A2 with six setae in the manuscript but with five in the figure.

####### Discussion.

The family Paramesochridae is divided into nine genera based on segmentation, and setal formula of swimming legs. Two new species clearly belongs to the genus *Emertonia*, because of: 1) one-segmented endopods of P2–P4 with one seta each, 2) three-segmented exopods of P2–P4, and 3) the one-segmented exopod of antenna. Kunz (1981) compared the width:length ratio of the caudal rami, the characteristics of caudal setae, and the number of setae on antenna, P4, and P5. Wells (2007) also considered the characteristics of caudal seta III, and the setae on P5, and the number and position of setae in antenna for identifying species of *Emertonia*.

Kunz (1981) and Huys (1987) proposed the phylogenetic position of the genera within the Paramesochridae. Kunz (1981) mentioned the diagnosis of *Kliopsyllus* based on the segmentation, and the seta formula of appendages. However, some species do not fit to the diagnosis by Kunz (1981). According to Huys’ (1987) cladogram of Paramesochridae, three genera in the *Paramesochra*-group are divided by six apomorphies: 1) four setae on distal segment of P1 exopod, 2) one-segemented endopod of P2–P4, 3) three setae on the distal segment of P4 exopod, 4) one-segmented exopod of P1, 5) P1 endopod without element, and 6) two setae on distal segment of P4 exopod and one seta on P4 endopod. However, some species of *Emertonia* do not fit to Huys’ cladogram as well. For example, *E. andeep* (Veit-Köhler, 2004) and *E. minor* (Vasconcelos, Veit-Köhler, Drewes & Parreira dos Santos, 2009) have two-segmented endopod of P4, *E. idiotes* (Wells, 1967) has three setae on P4 exp-3, and *E. gracilis* Wilson, 1932 and *E. pseudogracilis* Krishnaswamy, 1957 have one-segmented exopod of P1 (Table 1). Therefore the diagnosis of *Emertonia* needs to be amended as follows:

####### Amended diagnosis.

Paramesochridae. Body cylindrical, broad anteriorly, rather flattened; with distinct separation between prosome and urosome; rostrum small, fused at base. Operculum not developed. Caudal ramus with 6 or 7 setae, seta I small or obscure. Antennule 7- or 8-segmented in female. Antennary exopod 1-segmented with 1–5 setae. Mandible biramous; exopod 1-segmented with 2–4 setae; distal segment of endopod with several basally fused setae at apex. Maxilla with 3 endites on syncoxa, first endite bilobed; endopod 1- or 2-segmented. Maxilliped with elongate basis. P1 biramous, with 2-segmented endopod and 1-or 2-segmented exopod. P2–P3 biramous, with 2- or 3-segmented exopods and 1-segmented endopods; P4 biramous, with 1- or 2-segmented endopod and 2- or 3-segmented exopod.

Five distinctive groups within genus *Emertonia* can be recognized based on segmentation and setal formula in swimming legs: 1) *gracilis*-group, 1-segmented exopod of P1; 2) *laurentica*-group, 2-segmented exopod of P2–P4; 3) *andeep*-group, 2-segmented endopod of P4; 4) *coelebs*-group, P4 exp-2 with one inner seta; 5) *holsatica*-group, 2-segmented exopod of P1, 3-segmented exopod of P2–P4, 1-segmented endopod of P2–P4, and P2–P4 exp-2 without inner seta. However, more studies including the mouthparts, and the numbers and shapes of elements on the appendages will be necessary to confirm the phylogenetic relationships among the species of *Emertonia*.

### A taxonomic key for the worldwide species of *Emertonia* is constructed as follows. Unfortunately, *E. pontica* (Serban, 1959) is excluded from the key due to incomplete original description (Serban, 1959; Wells, 2007)

**Table:** 

1	P1 exopod 1-segmented…(*gracilis*-group)	**2**
–	P1–P4 exopod 2-segmented…(*laurentica*-group)	***E. laurentica***
–	P1 exopod 2-segmented , P4 endopod 2-segmented…(*andeep*-group)	**3**
–	P1 exopod 2-segmented, P4 exopod 3-segmented; P4 exp-2 with 1 inner seta (*coelebs*-group)	**4**
–	Theses character not combined…(*holsatica*-group)	**7**
2	P2–P3 exp-3 with 2 setae/spines	***E. gracilis***
–	P2–P3 exp-3 with 3 setae/spines	***E. pseudogracilis***
3	P4 enp-2 with 2 setae	***E. andeep***
–	P4 enp-2 with 1 seta	***E. minor***
4	P2–P3 exp-2 with 1 inner seta	***E. furcavaricata***
–	P2–P3 exp-2 without inner seta	**5**
5	Length of caudal rami 5 times as long as wide; P4 enp-1 with 2 setae	***E. coelebs***
–	These characters not combined	**6**
6	P4 enp-1 with 1 seta; P4 baseoendopodal lobe with 2 setae	***E. psammophila***
–	P4 enp-1 with 2 setae; P4 baseoendopodal lobe with 1 seta	***E. atlantica***
7	P2 exp-3 with 3 setae/spines	**8**
–	P2 exp-3 with 4 setae/spines	**14**
8	P1 exp-2 with 5 setae/spines	***E. minuta***
–	P1 exp-2 with 4 setae/spines	**9**
9	A2 exopod with 1 seta at most	***E. capensis***
–	A2 exopod with 2 setae at least	**10**
10	A2 exopod with 2 setae and P5 baseoendopodal lobe with 3 setae	***E. masryi***
–	These characters not combined…(*E. constrictus* s. str.)	**11**
11	A1 7-segmented and A2 exp with 3 setae at most	**12**
–	A1 8-segmented and A2 exp with 4 setae	**13**
12	P1 enp-1 length 1.6 times longer than P1 exp, length of caudal rami 3 times as long as wide	***E. constricta constricta***
–	P1 enp-1 length 1.2 times longer than P1 exp, length of caudal rami 2 times as long as wide	***E. constricta orotavae***
13	V-shaped baseoendopod of male P5 without setules	***E. constricta pacifica***
–	Each side baseoendopodal lobe almost fused ornamented with row of setules	***E. constricta egyptica***
14	P4 exp-3 with 3 setae	**15**
–	P4 exp-3 with 2 setae	**16**
15	A2 exp with 2 elements and P5 baseoendopod with 1 seta	***E. panamensis***
–	A2 exp with 3 elements and P5 baseoendopod with 2 setae	***E. idiotes***
16	P4 enp-1 with 2 setae	**17**
–	P4 enp-1 with 1 setae	**21**
17	P3 exp-3 with 3 setae	***E. perharidiensis***
–	P3 exp-3 with 4 setae	**18**
18–	Length of caudal rami more than 3.5 times as long as wide; penultimate somite normal	***E. holsatica varians***
–	Length of caudal rami more than 5 times as long as wide	**19**
19	Penultimate somite normal; endopod of P4 with 2 pinnate setae	***E. diva***
–	Penultimate somite elongated and ornamented 2 dorsal processes/spines; endopod of P4 with 1 bare and 1 brushlike setae	**20**
20	Length of caudal rami over 6–7 times as long as wide	***E. spiniger spiniger***
–	Length of caudal rami over 9 times as long as wide	***E. spiniger ornata***
21	P5 exopod with 5 setae	***E. pygmaea***
–	P5 exopod with 3 setae	**22**
22	Shape of caudal rami conical, sub-triangular	**23**
–	Shape of caudal rami square	**24**
23	A2 exopod with 4 setae; median depression between baseoendopodal lobes shallow	***E. acutifurcata***
–	A2 exopod with 2 setae; median depression between baseoendopodal lobes deeply	***E. koreana* sp. n.**
24	A2 exopod with 1 seta	***E. enalia***
–	A2 exopod with at least 2 setae	**25**
25	A2 exopod with 2 setae	**26**
–	A2 exopod with 3 setae	**28**
–	A2 exopod with 4 setae	**33**
–	A2 exopod with 5 setae	**38**
26	Caudal rami with inwardly pointed spine and long seta V	***E. longisetosa***
–	These characters not combined	**27**
27	End of P2–P3 enp globular; A2 exopod with 2 or 3 setae	***E. holsatica longicaudata***
–	Shape of P2–P3 endopods normal; A2 exopod with 2 setae	***E. arenicola***
28	Distal segment of P3 exopod with 3 setae	***E. major***
–	Distal segment of P3 exopod with 4 setae	**29**
29	Seta V of caudal rami consisted of two type elements, proximal half stout and distal half slender seta	***E. miguelensis***
–	Seta V of caudal rami normal	**30**
30	A1 8-segmented; P1 endopod 1.3 times longer than exopod; length of caudal rami twice as long as wide	***E. debilis***
–	These characters not combined	**31**
31	A1 7-segmented; P2 and P3 endopod with a single tiny spinule-like seta each; length of caudal rami 3 times as long as wide	***E. wilsoni***
–	These characters not combined	**32**
32	Baseoendopod of P5 with two apical setae; male exp of P5 with 3 setae	***E. californica***
–	Baseoendopod of P5 with one apical and one outer setae; male exp of P5 with four setae	***E. psammobionta***
33	Baseoendopodal lobes fused forming large plate; A1 8-segmented; male exp of P5 with four setae	***E. insularis***
–	These characters not combined	**34**
34	A1 8-segmented; P1 enp-1 ornamented with long setules; length of P1 enp-1 1.6 times as long as P1 enp-2; male P5 exp with three setae	***E. chilensis***
–	These characters not combined	**35**
35	Seta III of caudal rami blunt spine; apical seta of A2 exp stout comparison with other three setae; Caudal rami length approx. 2.5 times as long as wide	***E. longifurcata***
–	These characters not combined	**36**
36	Maxilliped enp 2-segmented; Caudal rami length approx. 1.6 times as long as wide	***E. brevicaudata***
–	These characters not combined	**37**
37	Caudal rami length approx. 2 times as long as wide; length of P1 enp-1 7 times as long as P1 enp-2	***E. holsatica holsatica***
–	Caudal rami length approx. 3 times as long as wide; length of P1 enp-1 approx. 2.7 times as long as P1 enp-2	***E. paraholsatica***
38	Baseoendopodal lobes fused forming large plate; P1 enp-1 and P1 exp equal in length	***E. regulexstans***
–	These character not combined	**39**
39	Caudal rami length more than 5 times as long as wide	**40**
–	Caudal rami length under 5 times as long as wide	**41**
40	Caudal rami length 9 times as long as wide; P5 exp with 1 pinnate and 2 bare setae, outermost longest	***E. schminkei***
–	Caudal rami length 5.5 times as long as wide; P5 exp with 3 bare setae, innermost longest	***E. clausi***
41	P2–P3 enp apical seta length longer than enp	**42**
–	P2–P3 enp apical seta length shorter than enp	**43**
42	P1 enp-2 with 1 seta	***E. ingridae***
–	P1 enp-2 with 2 setae/spine	***E. simplex* sp. n.**
43	Length of P1 enp-1 2.5 times as long as P1 exp; male P5 exp with 3 setae	***E. unguiseta***
–	P1 enp-1 and P1 exp same length; male P5 exp with 4 setae	***E. similis***

## Supplementary Material

XML Treatment for Emertonia koreana

XML Treatment for Emertonia simplex
